# Energy partitioning in cattle fed diets based on tropical forage with the inclusion of antibiotic additives

**DOI:** 10.1371/journal.pone.0211565

**Published:** 2019-04-22

**Authors:** Marcelina Pereira da Fonseca, Ana Luiza da Costa Cruz Borges, Pedro Henrique de Araujo Carvalho, Ricardo Reis e Silva, Lúcio Carlos Gonçãlves, Iran Borges, Helena Ferreira Lage, Alexandre Lima Ferreira, Eloísa Oliveira Simões Saliba, Diogo Gonzaga Jayme, Joana Ribeiro da Glória, Décio Souza Graça, Rodrigo Melo Meneses, Antônio Último de Carvalho, Elias Jorge Facury Filho, Arthur Alves Silva

**Affiliations:** Department of Animal Sciences, Veterinary School, Federal University of Minas Gerais, Belo Horizonte, MG, Brazil, Pampulha; University of Illinois, UNITED STATES

## Abstract

The aim of this study was to describe energy partitioning in dairy crossbreed bulls fed tropical forage-based diets supplemented with different additives. Twenty F1 crossbred bulls (Holstein x Gyr) with initial and final live weight (LW) averages of 190 ± 17 and 275 ± 20 kg were fed sorghum (*Sorghum bicolour*) and Tanzania grass (*Panicum maximum* cv. Tanzania) silage (70:30 DM basis) with supplemented concentrate at a forage to concentrate ratio of 50:50. The bulls were allocated to four treatment: control groups (without additives), monensin [22 mg/kg monensin dry matter (DM)] (M), virginiamycin (30 mg/kg virginiamycin DM) (V), and combination (22 mg/kg DM of monensin and 30 mg/kg DM of virginiamycin) (MV), in a completely randomised design. The intake of gross energy (GE, MJ/d), digestible energy (DE, MJ/d), metabolizable energy (ME, MJ/d), as well as energy losses in the form of faeces, urine, methane, heat production (HE), and retained energy (RE) were measured. Faecal output was measured in apparent digestibility trial. Right after the apparent digestibility trial, urine samples were collected in order to estimate the daily urinary production of the animals. Heat and methane production were measured in an open circuit respirometry chamber. The intake of GE, DE, and ME of the animals receiving monensin and virginiamycin alone or in combination (MV) showed no differences (P>0.05) from the control treatment. However, the MV treatment reduced (P<0.05) the methane production (5.44 MJ/d) compared to the control group (7.33 MJ/d), expressed in MJ per day, but not when expressed related to gross energy intake (GEI) (CH_4_, % GEI) (P = 0.34). Virginiamycin and monensin alone or in combination did not change (P>0.05) the utilization efficiency of ME for weight gain, RE and net gain energy. This study showed that for cattle fed tropical forages, the combination of virginiamycin and monensin as feed additives affected their energy metabolism by a reduction in the energy lost as methane.

## Introduction

The energy partitioning process consists of evaluating the amount of energy ingested by the animal and quantifying the loss of this energy during metabolism. Research has shown that for ruminants between 6 and 12% of gross energy (GE) intake is lost in the form of methane [1].

Improving the efficiency of the rumen fermentation is a long-time process for ruminant nutritionists–especially finding strategies to mitigate methane production. Ionophores are a class of compounds used to improve this efficiency with considerable success as a feed additive and continue to be the subject of some studies [2]. Monensin, anionophore produced by strains of *Streptomyces cinamonensis*, was recorded in 1975 as a feed efficiency enhancer for confined cattle and is currently one of the most widespread feed additives for cattle and poultry.

Although in 2006 the European Union banned feeding ionophores to food producing animals [3], its use has approval as cattle feed in both Canada and the USA [4].

Its best documented effects are improvement in the efficiency of rumen fermentation, reduction in methane production, and inhibition of dietary protein degradation in the rumen [5], [4] (. Ionophores are molecules with a diverse chemical structure, including monensin, acting to disrupt the transmembrane movement and intracellular equilibrium of ions in certain bacteria and protozoa in the ruminant digestive tract.

Virginiamycin, a non-ionophore antibiotic derived from *Streptomyces virginiae* which reduces the growth of Gram-positive bacteria by binding to ribosomes and inhibiting peptide synthesis. Moreover, inhibition of the growth of lactic acid producing bacteria ([6], [7] and improved post-ruminal nutrient uptake [8] have also been reported.

The effect of monensin and virginamycin on energy efficiency is related to its ability to selectively inhibit gram-positive over gram-negative bacteria that reduce succinate to propionate. Increased propionate to acetate ratios and reduced numbers of protozoa-generating hydrogen. Currently, there is limited data on the effects of feeding monensin and/or virginamycin to cattle fed tropical-based diets [9]. There are no data related to energy partitioning in crossbreed cattle in tropical conditions feeding additives, and this study is pioneer to quantify this data.

The two additives have different action mechanisms, virginiamycin is a bactericide antibiotic, that disrupting the bactéria metabolism. Monensin are a bacteriostatic antibiotic, selecting the same types of bacteria as virginiamycin, but in a different way. The association between bactericide and bacteriostatic antibiotics is not recommended in a therapeutic way, since inhibition of the action of both antibiotics may occur. However, the association of these additives with the aim of rumen modulators may occur due to the large number of bacteria in the rumen and the low dose used.

The aim of this study was to describe the energy partitioning in cattle fed tropical foragebased diets supplemented with different additives of monensin and/or virginiamycin.

## Materials and methods

### Study site, animals, diets, and experimental design

The experimental protocol followed the guidelines for the use of animals for scientific purposes in Brazil and was approved by the Ethics and Animal Experimentation Committee of the Federal University of Minas Gerais, under protocol 215/10.

The experiment was conducted at the Animal Metabolism and Calorimetry Laboratory (LAMACA) of the Department of Animal Sciences at the School of Veterinary Medicine, Federal University of Minas Gerais, Belo Horizonte, state of Minas Gerais, Brazil.

Twenty F_1_ bulls (Holstein x Gyr) with average initial age of eight months and initial and final live weight averages of 190 ± 17 and 275 ± 20 kg, respectively, were allocated to four treatments in a completely randomised design. The treatments were: control (no additives), monensin (22 mg monensin/kg DM concentrate), virginiamycin (30 mg virginiamycin/kg DM concentrate), and combination (monensin and virginiamycin, 22 and 30 mg/kg DM concentrate, respectively).

The monensin and virginiamycin additives were included in the concentrate under the commercial brands Rumensin®100 (10% concentration) produced by Elanco (Greenfield, IN, USA) and Eskalin® (2% concentration) produced by Phibro (Ridgefield Park, NJ, USA), respectively.

The diets were formulated according to the [10] recommendations in order to meet cattle requirements of 1.0 kg live weight gain (LWG) and were isonitrogenous and isocaloric. We used silage made from sorghum (*Sorghum bicolor* (L.) and Tanzania grass (*Panicum maximum cv* Tanzania) and concentrate made of corn, soybean meal, urea, salt, and a mineral core. The sorghum and Tanzania grass were grown together at the same area and reaped 90 d after planting, with sorghum grains in the milky doughy phase. The sorghum and Tanzania grass were ensiled together. The proportions of sorghum and Tanzania grass in the silage were 70 and 30%, respectively. The forage concentrate ratio was 50:50, based on DM, and remained fixed throughout the experiment.

The experiment lasted for 70 d until completion, consisting of a 44-d adaptation and intake stabilization and rumem adaptation to additives followed by a 5-d total faeces collection period, 1-d urine collection, before the animals were staged over 20 d into an individual respirometric chamber. One animal from each treatment had its measurements taken daily in the respirometric chamber, until all the animals passed through the equipment, completing the 20 d [11]. The sequence of animals to had respirometric chamber measurements was related to additives group. One animal of each group had the data measured to ensure that the day of data collection will not influence in the result in any group.

Diet was provided twice a day, at 9 AM and 5 PM. Before feeding, the forage was mixed with the concentrate to ensure complete dietary intake and to avoid any food selection by the animals. To ensure *ad libitum* intake for 1.0 kg ADG without selection by animals, food intake was adjusted daily so that orts were 10%. As a mineral source, the mineral core with trade name Core 160 ®, produced by Alvorada (Maravilhas, Minas Gerais, Brazil), was used. The proportion of the ingredients and composition of the experimental diets are shown in Table 1.

**Table 1:** Ingredients and chemical composition of the experimental diets.

		Treatment^b^ (g/kg DM)		
	C	M	V	MV
Silage^a^	500.0	500.0	500.0	500.0
Soybean meal	98.9	98.9	98.9	98.9
Ground corn	372.3	372.3	372.3	372.3
Urea	7.2	7.2	7.2	7.2
Salt (NaCl)	2.3	2.3	2.3	2.3
Core 160	19.3	19.3	19.3	19.3
	Chemical composition (g/kg DM)			
DM (g/kg)	566.7	568.3	566.2	566.8
OM	943.5	937.6	941.1	939.4
Ash	56.5	62.4	58.9	60.6
CP	165.0	159.5	162.5	164.5
aNDFom	369.5	358.5	372.9	364.8
ADF	203.5	203.6	205.2	205.3
NFC	381.2	390.8	380.6	377.3
EE	20.8	21.5	22.0	22.2

^a^300 g/kg Tanzania grass and 700 g/kg sorghum.

^b^C, control diet; M, diet with monensin (22 mg kg/ DM of monensin)

V, diet with virginiamycin (30 mg/kg DM of virginiamycin); MV, diet with both additives ((22 mg kg/ DM of monensin and 30 mg/kg DM of virginiamycin). Core 160 (composition/kg): 250 g of calcium, 160 g of phosphorus, 30 g of sulfur, 30 g of magnesium, 200 mg of cobalt, 2500 mg of copper, 160 mg of iodine, 2100 mg of manganese,9000 mg of zinc, 40 mg of selenium and 1700 mg of fluorine.

### Facilities and management

The animals were ear tagged, vaccinated and dewormed prior to the start of the experiment. The animals were kept confined in a tie-stall type barn with a concrete floor. In order to provide greater comfort, each stall was equipped with VEDOVATI® perforated rubber pallets with the following dimensions: 1.10 m long, 0.90 m wide and 0.1 m thick. The daily floor cleaning procedures involved the complete removal of faeces and urine followed by thorough washing. A trough and a drinking fountain were available for each animal.

### Weight gain measurements

The animals were weighed every 15 days on two consecutive days around 8 AM, immediately before the morning feeding, and the mean of the two weighing results was used.

### Total faecal and urine collection

The total faecal production, urine production and respirometric measurements was held on consecutive days. Daily faecal production was measured by total faeces collection for five consecutive days (120 hours) in the tie stall immediately before the urine collection and respirometric measurements. The faeces were immediately collected and placed in individual containers for each animal.

Once the feed given, orts and faeces were collected, weighed, and sampled twice a day. Samples of approximately 300 to 400 grams were placed in plastic bags and frozen in a Freezer at -15°C for later analysis. The total faecal production was measured twice a day to determi the faecal volum.

Samples of food, orts and faeces were pre-dried at 55°C for 72 hours in a ventilated oven, then ground in a Thomas-Willey type stationary grinder equipped with a 5 mm sieve, to create the composite samples. The faeces composites were made of faeces samples collected from each animal during the digestibility test. Subsequently, each composite sample and the individual samples were ground again, in a stationary grinder equipped with a sieve having 1 mm strainers.

Right after the 5-d total fecal collection, urine spot samples were collected 4 hours after feeding, during spontaneous urination. An aliquot of 60 mL of urine was collected to determine creatinine, nitrogen, and gross energy concentrations. The urine volume was estimated by multiplying the LW by the daily creatinine excretion in mg/kg of LW and dividing the product by the concentration of creatinine (mg/L) in the urine, according to [12] (. To determine the daily creatinine excretion per kilogram of LW, an average of 28.72 mg/kg.

LW was used, as determined by [13] for Holstein x Gyr crossbred cattle.

### Methane and heat production measurements

Methane and heat production were measured over 22 to 23 hours by performing a 24 hours extrapolation period, using an open-circuit breathing chamber, adopted by the UFMG Veterinary School, according to [11]. The respirometric chamber was made of steel and had acrylic side windows, 3.45 m long, 1.45 m wide, and 2.45 m high (22.391 L of internal volume).

In this system, the animal is housed in a chamber with a sealing that does not allow any gas exchange with the outside air, except by a proper air circulation system. The air present inside the chamber is continuously renewed by the constant intake of external air. The intake of fresh air into the chamber is possible due to the negative pressure created inside by the pump that promotes the suction of the internal air, thus allowing the entrance of external air. As a consequence, the internal atmosphere of the chamber is renewed and the air contained can be destined for sampling and later evaluation by the gas analyzers (oxygen consumption, carbon dioxide and methane production).

### Feed, faeces and urine analysis

Samples of feed, orts, and faeces were defrosted at room temperature and pre-dried at 55 ± 5°C for 72 hours [14]. Feed and faecal samples collected over the experimental duration were individually ground in a stationary Thomas-Willey mill using a 5mm mesh sieve before a composite sample for each individual animal’s feed and faeces was made. The composite samples were further ground using a 1mm sieve and stored in polyethylene flasks for chemical analyses.

The DM content was determined at 105°C (proc. 930.15; [15]). The content of organic matter (OM) was calculated as the difference between DM contents and ash content, with ash content determined by combustion at 600°C for 4 hours (proc. 935.05; [15]) The crude protein content (CP, 6:25 x nitrogen) was measured according to the Kjeldahl method (proc. 976.05; [15]) and the ether extract (EE) according to the Soxhlet method (proc. 963.15;[15]).

The neutral detergent fibre content (aNDFom) and acid detergent fibre (ADF) were determined in a Fibre Analyser ANKON® device (AnkomTM technology, Fairport, NY, USA) by serial method, as described by [16]. For the aNDFom procedure, 500 μL/g DM of heat-stable amylase (Termamyl 2x) by Novozymes Latin America Ltda (Araucaria, Parana, Brazil) were used, expressed exclusive of residual ash. Analyses of aNDFom and ADF were performed using 5x5 cm sachets made of nonwoven fabric with 100 micron porosity [16] (Van Soest, 1994). The nonfibrous carbohydrates (NFC) were calculated according to the equation proposed by [17] as follows: 100 - [(% CP—%CP urea +% urea) +% aNDFom +% EE +% ash].

Urine samples were stored in sealed plastic pots; a sample aliquot was stored at the ratio of 1 part urine to 9 parts 40% sulfuric acid for subsequent analysis of creatinine levels [12]. Another aliquot was stored *in natura* to assess N and GE levels. They were both frozen in a cold chamber for future analysis. Analyses of creatinine concentration in urine were carried out in the Clinical Pathology Laboratory of the UFMG Veterinary School, using COBAS® equipment. The total N was determined by the Kjeldahl method (proc. 976.05; [15]).

The GE was determined by combustion in an adiabatic bomb calorimeter PARR 2081 model, for the feed, orts, faeces, and urine samples.

### Calculations

Energy partitioning was determined by subtracting the energy losses in the faeces, urine, methane, and the daily heat production from the GE intake. The DE was determined by subtracting energy losses in form of feces from GE. The ME was determined by subtracting energy losses in form of methane and urine from DE. The energy loss in the form of methane was quantified in the fed animals, assuming a loss of 9.45 kcal/L of produced methane, according to [18]. The DE and ME concentrations of the diet were calculated using the ratio between energy intake and DM intake. The diet metabolisability was calculated from the ratio between the ME and the GE intake [19].

The daily heat production of the animals was determined using the equation proposed by [18]: HP (kJ) = 16.18 O_2_ + 5.02 CO_2_−2.17 CH_4_−5.99 N. Where O_2_ is the volume of consumed O_2_ (L/d), CO_2_ is the volume of produced CO_2_ (L/d), CH_4_ is the volume of produced CH_4_ (L/d), and N the amount of nitrogen excreted in the urine (g/d).

Retained energy (RE) was obtained by subtracting HP from MEI. The partial efficiency of the use of ME for gain (k_g_) was calculated as the coefficient of the slope of the linear regression of RE as a function of MEI, according to the following model: RE = β_0 + (_β_1_ x MEI). where RE = retained energy (MJ/kg BW_0.75_/d), MEI = metabolizable energy intake (MJ/kg BW^0.75^/d), and β_0_ and β_1_ are regression parameters. Under this model, *β*_1_ represents the k_g_.

### Statistical analysis

The experiment was conducted using a completely randomised design with four treatments and five animals by treatment. Each animal represented an experimental unit, as per the statistical model: Y ij = M + Ti + eij, where M = overall average, Ti = treatment effect, and eij = random error associated with the observations. The variables were subjected to variance analysis (ANOVA) using the SAS software [20], version 8.0. Differences between treatments were considered significant at P<0.05 using the Tukey test for comparisons between means.

## Results

The energy concentration of the diets, average daily weight gain, and feed efficiency were not affected (P>0.05) by the treatments (Table 2).

**Table 2:** Energetic concentration of the experimental diets, performance and feed efficiency in F1 Holstein x Gir bulls feeding diet with inclusion of monensin, virgiamycin or both.

	Treatment						
	C	M	V	MV	Mean	SEM^a^	Pvalue
GE (MJ/kg)	16.3	16.2	16.2	16.3	16.3	0.0413	0.2581
DE (MJ/kg)	10.7	10.7	10.7	10.8	10.8	0.1601	0.9381
ME (MJ/kg)	9.28	9.53	9.41	9.33	9.38	0.2021	0.8636
NEg (MJ/kg)	2.21	2.92	2.38	2.52	2.5	0.3310	0.5792
ADG (kg/day)	1.54	1.44	1.38	1.29	1.41	0.0731	0.1449
FE (Kg LW/kg DM)	0.216	0.200	0.205	0.207	0.207	0.0052	0.2950

^a^ SEM, standard error of the mean, n = 20.

C, control diet; M, diet with monensin; V, diet with virginiamycin MV, diet with both additives. GE, gross energy; DE, digestible energy; ME, metabolizable energy; NEg, net energy for gain; ADG, average daily gain; FE, feed efficiency (live weight / kg of Dry matter intake).

There was no difference in the intake of GE (P = 0.1908) and DE (P = 0.3170) between the treatments, which averaged 111 MJ/d and 74.0 MJ/d, respectively (Table 3). The loss in faecal 258 GE (MJ/d) did not differ (P = 0.1523) between the control and the additive treatments.

**Table 3:** Energy partitioning of F1 Holstein x Gir bulls feeding diet with inclusion of monensin, virgiamycin or both.

	Treatment					
	C	M	V	MV	SEM	Pvalue
DMI (kg/d) GEI (MJ/d)	7.1 117	7.2 117	6.7 109	6.2 101	1.121 5.595	0.1922 0.1908
Fecal GE (MJ/d)	40.1	40.0	35.5	34.3	2.100	0.1523
Fecal GE (%GEI)	34.18	33.92	32.79	33.68	1.238	0.8701
DEI (MJ/d)	77.07	77.74	73.94	67.54	4.127	0.3170
Urine GE (MJ/d)	3.35	2.81	3.28	3.96	0.7440	0.7529
Urine GE (%GEI)	2.80	2.38	3.01	3.91	0.5790	0.3286
Methane GE (MJ/d)	7.33	6.08^a^^b^	6.78^a^^b^	5.44^b^	0.4204	0.0299
Methane GE (%GEI)	6.31	5.17	6.22	5.37	0.3958	0.1342
MEI (MJ/d)	66.3	68.8	63.8	58.1	3.792	0.2617
q (ME/GE)	0.57	0.59	0.58	0.57	0.0132	0.7650
k_g_	0.242	0.309	0.253	0.268	0.0351	0.5687
ME/DE	0.86	0.88	0.86	0.86	0.0094	0.2038
HP (MJ/d)	49.6	47.6	47.3	42.2	2.256	0.1603
RE (MJ/d)	16.7	21.2	16.5	15.9	2.868	0.5537
RE (%GEI)	13.7	18.0	14.8	15.4	2.200	0.5682

Averages followed by different letters on the row are statistically different by the Tukey test (P<0.05).

^a^SEM, standard error of the mean, n = 20.

^b^C, control diet; M, diet with monensin (22 mg kg/ DM of monensin); V, diet with virginiamycin (30 mg/kg DM of virginiamycin); MV, diet with both additives ((22 mg kg/ DM of monensin and 30 mg/kg DM of virginiamycin)DMI, dry matter intake; GEI, GE intake; DEI, DE intake; MEI, ME intake; q, diets metabolisability; k_g_, utilization efficiency of the ME for gain; ME/DE, ratio between ME and DE; HP, daily heat production; RE, retained energy.

The energy lost as urine (MJ/d and % GE) did not differ between treatments (P = 0.3286).

Animals on the combined monensin and virginamycin treatment lost less (P = 0.03) methane energy (MJ/d), compared to the control treatment. On the other hand, methane production as a percentage of GE intake was not significantly different (P = 0.1342) between the treatments, 5.76% on average (Table 3). The ME intake showed no difference (P = 0.2617) between the groups and averaged 64.3 MJ/d.

The metabolisability and the relationship between ME and DE of the diets did not change (P = 0.2038). The retained energy was not significantly different (P = 0.5537) between the treatment groups, with an average value of 17.6 MJ/d. The utilization efficiency of the ME for weight gain was not significantly different (P = 0.5692) and averaged 0.268.

## Discussion

This study describes the energy partitioning of tropical forage-based diets supplemented with different additives and fed to cattle. [5] found no effect (P>0.05) of monensin on the GE intake in cattle, similarly as ours results. There was no difference in GE intake since no difference was observed in dry matter intake (DMI). Approximately 30% of the consumed GE is lost in the form of faeces [1], [21]. In the present study, the energy lost as faecal output was, on average, 33.6%.

The production of methane (MJ/d) varied from 5.44 to 7.33 MJ/d and was significantly reduced with the combined use of monensin and virginiamycin, compared to the control treatment. According to [22], a reduction in DM intake associated with the use of monensin alone can represent a reduction of up to 55% in methane production in animals fed forage-based diets. In our work, the DM intake was not statistically altered [23].

It is well established that monensin is effective against Gram-positive, acetate producing bacteria, such as *Ruminococcus* and *Butyrivibrio* [24]. Its effect on this group of bacteria is associated to the higher concentration of nicotinamide adenine dinucleotide (NADH/NAD^+^), which favours propionate synthesis by NADH re-oxidation, resulting in limiting the amount of hydrogen (H_2_) available for methane synthesis by the Archaea group [25], [26].

The energy lost as methane production in relation to consumed GE was similar to that achieved by [27]. The value reported by these authors was 5.91%, for calves receiving 33 mg/kg DM monensin. Reduction in methane production through the use of monensin was also confirmed by [28] in calves fed Rhodes grass (*Chloris gayana*) hay with addition of 240 mg/d monensin. According to these authors, methane production was reduced from 76.9 g/d or 4.4% of consumed GE to 48.1 g/d or 3.02% of GE consumed between the control and the monensin treatments, respectively. Previously studies using monensin combined diets based on conserved forage and concentrate in cattle reported reductions in methane production of up to 30% [27], [5].

Urinary excretion of energy is closely associated with N urinary excretion [29]. Amino acids and creatinine have a higher energy content than urea, which represents 80 to 90% of urinary N.

Virginiamycin and monensin had no effect on ME intake, compared to the control treatment. Possibly, the improved efficiency of ME use, which usually occurs with the use of these compounds, was not observed due to the type of diet, which was in this case based on sorghum silage with tropical grass. The faecal fraction has a major part on energy partitioning, compared to the losses in gases and urine. Since faecal energy loss was not altered and the ME is calculated by deducting the energy losses of urine and methane from the DE, this result is consistent. [30] registered a reduction in ME intake (79.0 MJ/d) with the combined use of salinomycin and virginiamycin, when compared to isolated use of salinomycin (87.8 MJ/d) in finishing cattle fed diets with high or low concentrate. The values reported by these authors are higher than those obtained in this study, probably due to the larger amount of grains used in their diets with a 73% or 91% concentrate. The average ME intake registered in this study (64.2 MJ/d) is within the range suggested by [31] for non-castrated crossbred zebu bulls, with a weight gain of 1.25 kg/d and an average weight between 200 and 250 kg.

The metabolizability of the diet depends on the diet quality. In Friesian and Holstein heifers fed diets with 10.03 MJ/kg DM of ME, the metabolizability of the diet was approximately 0.53 [19]. The [10] states that the relationship between ME and DE for beef cattle is 0.80. The relationship between the ME and DE average of this study (0.86) is, however, within the range established by the British feeding system [19] which ranges between 0.81 and 0.86. These results are important, because our study is pioneer to quantify the energy partitioning in crossbreed cattle in tropical conditions.

The similarity in the retained energy registered in our study is in line with [32], who also observed no effect of monensin on energy retention in Hereford steers fed corn silage and concentrate. The average value recorded by that author was 17.6 MJ/d, quite similar to the one obtained in this work (17.5 MJ/d).

Unlike the present study, [8] observed an increase in the net energy concentration estimated for maintenance and weight gain for diets supplemented with virginiamycin for steers in the growing and finishing stages. In contrast, monensin, as in our study, had no effect.

The [10] is based on a confined cattle database, where ionophore is usually used. According to this Committee, the net energy requirement for weight gain in cattle with a LW between 200 and 250 kg for a weight gain of 1.5 kg/d is between 17.5 and 20.9 MJ/d. In our study, the net energy for weight gain, equivalent to 17.5 MJ/d, is within the range suggested by the U.S system for this weight range.

Compared to the control treatment, the additives used in this study were not effective in changing cattle daily LWG or feed efficiency. This can be attributed to the difference between breed and significant genetic potential of beef cattle, which are used in most studies involving the use of these compounds, compared to the animals of this study, which are derived from animals with a dairy origin. Consistent with this result, the diets’ net energy for weight gain, as well as the use efficiency of the ME for weight gain, were similar between the groups. [33] also found no difference in the daily average weight gain and feed efficiency of cattle fed virginiamycin combined with salinomycin in relation to those treated with these additives singularly.

The [10] postulates that the utilisation efficiency of ME for weight gain varies between 0.29 and 0.47, considering feeds with ME concentrations of 8.3 and 13.3 MJ/kg DM, respectively. The average value in this study (0.27) is close to the minimum suggested value. According to [34], the ratio of energy directed for protein or fat synthesis is determinant for the variation of the utilization efficiency of ME for weight gain, since the deposition of fat is more energy-efficient (60 to 80%) than that of protein (10 to 40%). The lower value for the utilization efficiency of ME for weight gain in the present study can be justified considering the normal growth curve for cattle. Animals in the early stages of growth, such as those used in this study, have lower utilization efficiency of ME for weight gain, possibly due to a higher protein deposition in that stage of development. This efficiency is highly related to the quality of the diet and therefore differences between diets must be considered. In this study, the diets were based on a silage made from sorghum and tropical grass, whose average ME content (9.36 MJ/kg DM) is probably lower than that used in the studies that gave rise to the [10], which are usually based on corn silage.

## Conclusions

This study showed that virginiamycin and monensin at doses of 30 mg/kg DM and 22 mg/kg DM, respectively, did not have a significant effect on the net energy for weight gain when feeding animals using sorghum and tropical grass silage-based diets.

The metabolizability (ME/GE) did not differ between the treatments, and averaged 0.58. The NE_g_ in cattle fed tropical forage was 2.5 MJ/kg.

The monensin association with virginiamycin showed potential benefits associated to the energetic metabolism of cattle, due to a reduced methane energy output, without difference in DMI. The methane production in the control treatment was 34% higher than in the treatment with monensin and virginiamycin.

## Supporting information

S1 Data This is raw data set used to reach the conclusions drawn in a manuscript. (XLSX)

## References

[1] Johnson D.E., Ferrell C.L., Jenkins T.G., 2003 The history of energetic efficiency research: Where have we been and where are we going? J. Anim. Sci. 81, 27–38. 10.2527/2003.8113_suppl_1E27x

[2] Bretschneider G., Elizalde J.C., Pérez F.A., 2008 The effect of feeding antibiotic growth promoters on the performance of beef cattle consuming forage-based diets: A review. Livest. Sci. 114, 135–149. 10.1016/j.livsci.2007.12.017.

[3] Tomkins N.W., Denman S.E., Pilajun R., Wanapat M., Mcsweeney C.S., Elliott R., 2015 Manipulating rumen fermentation and methanogenesis using an essential oil and monensin in beef cattle fed a tropical grass hay. Anim. Feed Sci. Technol. 200, 25–34. 10.1016/j.anifeedsci.2014.11.013.

[4] Erasmus L.J., Muya C., Erasmus S., Coertze R.F., Catton D.G., 2008 Effect of virginiamycin and monensin supplementation on performance of multiparous Holstein cows. Livest. Sci. 119, 107–115. 10.1016/j.livsci.2008.03.005

[5] Guan H., Wittenberg K.M., Ominski K.H., Krause D.O. 2006 Efficacy of ionophores in cattle diets for mitigation of enteric methane. J. Anim. Sci. 84, 1896–1906. 10.2527/jas.2005-652 16775074

[6] Nagaraja T.G., Taylor M.B., Harmon D.L., Boyer J.E., 1987 *In vitro* acid inhibition and alterations in volatile fatty acid production by antimicrobial feed additives. J. Anim. Sci. 65, 1064–1076. 3667452 10.2527/jas1987.6541064x

[7] Clayton E.H., Lean I.J., Rowe J.B., Cox J.W., 1999 Effects of feeding virginiamycin and sodium bicarbonate to grazing lactating dairy cows. J. Dairy Sci. 82, 1545–1554. 10.3168/jds.S0022-0302(99)75382-8 10416170

[8] Salinas-Chavira J., Lenin J., Ponce E., Sanchez U., Torrentera N., Zinn R.A., 2009 Comparative effects of virginiamycin supplementation on characteristics of growthperformance, dietary energetics, and digestion of calf-fed Holstein steers. J. Anim. Sci. 87, 4101–4108. 10.2527/jas.2009-1959 19749020

[9] McGuffey R.D., Richardson L.F., Wilkinson J.I.D., 2001 Ionophores for dairy cattle: current status and future outlook. J. Dairy Sci. 84, 194–203. 10.3168/jds.S0022-0302(01)74469-4 11210033

[10] NRC, 2000. Nutrient requirements of beef cattle, 7th revised. ed National Academy Press, Washington, DC, (2000)

[11] SILVA R. R. E.; BORGES A. L. C. C.; Carvalho P.H.A.; SOUZA A. S.; VIVENZA P. A. D.; SILVA J. S.; et al Respirometry and Ruminant Nutrition. Animal Husbandry and Nutrition 1ed Londres: IntechOpen, 2018, v. 1, p. 171–190.

[12] Valadares R.F.D., Broderick G.A., Valadares Filho S.C., Clayton M.K., 1999 Effect of replacing alfalfa with high moisture corn on ruminal protein synthesis estimated from excretion of total purine derivatives. J. Dairy Sci. 8, 2686–2696 10.3168/jds.s0022-0302(99)75525-6 10629816

[13] Rennó L.N., Valadares Filho S.C., Paulino M.F., Leão M.L., Valadares R.F.D., Rennó F.P., et al, 2008 Níveis de ureia na ração de novilhos de quatro grupos genéticos: parâmetros ruminais, ureia plasmática e excreções de ureia e creatinina. Rev. Bras. Zootec. 37, 556–562

[14] Silva DJ, Queiroz AC 2006 Análise de alimentos (métodos químicos e biológicos). 4th edn Editora UFV Viçosa (2006).

[15] AOAC, 1990. AOAC, Official Methods of Analysis, (15th ed), Int., Gaithersburg, MD (1990)

[16] Van Soest P.J., Robertson J.B., Lewis B.A., 1991 Methods for dietary fiber, neutral detergent fiber and non-starch polysaccharides in relation to animal nutrition. J. Dairy Sci. 74, 35833597 10.3168/jds.S0022-0302(91)78551-2. 1660498

[17] Hall M.B., 2000 Neutral detergent-soluble carbohydrates. Nutritional relevance and analysis, a laboratory manual. Gainesville: University of Florida. (Extension Bulletin, 339). 42p.

[18] Brouwer, E., 1965. Report of sub-committee on constants and factors. In: Symposium of Energy Metabolism Held at European Association for Animal Production. EAAP Academic, London, pp. 441–443.

[19] Agricultural and Food Research Council, 1993. Energy and requirements of ruminants, Commonwealth Agricultural Bureaux International, Wallingford, UK.

[20] SAS, 1999 SAS/STAT User’s Guide (Version 8.01th ed.). SAS Institute Inc., Cary, NC.

[21] Ferrell C.L., Oltjen J.W., 2008 ASAS Centennial Paper: Net energy systems for beef cattle concepts, application, and future models. J. Anim. Sci. 86, 2779–2794. 10.2527/jas.20080954 18820167

[22] O’Kelly J.C., Spiers W.W., 1992 Effect of monensin on methane and heat production of steers fed lucerne hay. Aust. J. Agric. Res. 43, 1789–1793.

[23] Fonseca M.P., Borges A.L.C.C., Silva R.R., Lage H.F.; lima A.F., lopes F.C.F., et al 2015 Intake, apparent digestibility, and methane emission in bulls receiving a feed supplement of monensin, virginiamycin, or a combination. Anim. Prod. Sci. online version. 10.1071/AN14742

[24] Appuhamy J.A.D.R.N., Strathe A.B., Jayasundara S., Wagner-Riddle C., Dijkstra J., France J., kebreab E., 2013 Anti-methanogenic effects of monensin in dairy and beef cattle: a metaanalysis. J. Dairy Sci. 96, 5161–5173. 10.3168/jds.2012-5923 23769353

[25] Rivera A.R., Berchielli T.T., Messana J.D., Velasquez P.T., Franco A.V.M., Fernandes L.B., 2010 Fermentação ruminal e produção de metano em bovinos alimentados com feno de capim-tifton 85 e concentrado com aditivos. Rev. Bras. Zootec. 39, 617–624. 10.1590/S1516-35982010000300022

[26] Golder H.M., Celi P., Rabiee A.R., Lean I.J. 2014 Effects of feed additives on rumen and blood profiles during a starch and fructose challenge. J. Dairy Sci. 97, 985–1004. 10.3168/jds.2013-7166 24210482

[27] McGinn S.M., Beauchemin K.A., Coates T., Colombatto D., 2004 Methane emissions from beef cattle: effect of monensin, sunflower oil, enzymes, yeast and fumaric acid. J. Anim. Sci. 82, 3346–3356. 10.2527/2004.82113346x 15542482

[28] Tomkins N.W., Denman S.E., Pilajun R., Wanapat M., Mcsweeney C.S., Elliott R., 2015. Manipulating rumen fermentation and methanogenesis using an essential oil and monensin in beef cattle fed a tropical grass hay. Anim. Feed Sci. Technol. 200, 25–34. 10.1016/j.anifeedsci.2014.11.013.

[29] Vasconcelos A.M., Leão M.I., Valadares Filho S.C., Valadares R.F.D., Dias M., Moraes D.A.E.F. 2010 Ruminal parameters, nitrogen compound balance and microbial production in dairy cows fed soybeans and their by-products. R. Bras. Zootec. 39. 425–433, 10.1590/S1516-35982010000200028.

[30] Nuñez A.J.C., Caetano M., Berndt A., Demarchi J.J.A.A., Leme P.R., Lanna D.P.D., 2013 Combined use of ionophore and virginiamycin for finishing Nellore steers fed high concentrate diets. Sci. Agric. 70, 229–236. 10.1590/S0103-9016201300040000290162013000400002.

[31] Valadares Filho S.C., Marcondes M.I., Chizzotti M.L., Paulino P.V.R. Exigências nutricionais de zebuínos puros e cruzados: BR-CORTE 2010 2ed Editora UFV Viçosa.

[32] Byers F.M., 1980 Determining effects of monensin on energy value of corn silage diets for beef cattle by linear or semi-log methods. J. Anim. Sci. 51, 158–169 7410268 10.2527/jas1980.511158x

[33] Silva S.L. Almeida R., Schwahofer D., Leme P.R., Lanna D.P.D., 2004 Effects of salinomycin and virginiamycin on performance and carcass traits of feedlot steers. J. Anim. Sci. 82, 41–42.14753347

[34] Freitas J.A., Queiroz A.C., Dutra A.R., Vieira R.A.M., Lana R.P., Leonel, et al 2006 Body composition and net energy requirements for maintenance of feedlot purebred and crossbred Nellore young bulls. Rev. Bras. Zootec. 35, 878–885. 10.1590/S1516-35982006000300034.

